# Novel non-coding RNA-based therapeutic approaches to prevent statin-induced liver damage

**DOI:** 10.1002/emmm.201201565

**Published:** 2012-08-20

**Authors:** Claudia Bang, Thomas Thum

**Affiliations:** 1 Institute of Molecular and Translational Therapeutic Strategies (IMTTS), Hannover Medical SchoolHannover, Germany; 2 Centre for Clinical and Basic Research, IRCCS San RaffaeleRome, Italy

**Keywords:** cholesterol, hepatotoxicity, miRNAs, statins, sterol transporters

See related article in EMBO Molecular Medicine http://dx.doi.org/10.1002/emmm.201201228

MicroRNAs (miRNAs) are tiny non-coding ribonucleic acids (RNAs) that control gene expression of target messenger RNA (mRNAs) at the posttranscriptional level by mRNA degradation or translational repression. Thus, miRNAs have emerged as critical regulators during physiological and pathological processes (Thum et al, 2007). Recently, several reports have revealed an important role for miRNAs in cholesterol and fatty acid homeostasis and also insulin signalling (Davalos et al, 2011; Marquart et al, 2010; Najafi-Shoushtari et al, 2010; Rayner et al, 2010, 2011a).

Cholesterol is a precursor in metabolic pathways and an important structural component in the cell membrane of most vertebrates. Notably, dysfunction in cholesterol homeostasis is linked to metabolic syndrome, atherosclerosis and type-2 diabetes (Rayner et al, 2011b). In general, the cholesterol metabolism is a complex process which is regulated mainly by three transcription factors; sterol regulatory element binding protein (SREBP), liver X receptor (LXR) and Farnesoid X receptor (FXR) (Beaven & Tontonoz, 2006). Further, it has been recently shown that also miRNAs particularly miR-33 posttranscriptionally control cholesterol metabolism (Marquart et al, 2010; Rayner et al, 2010, 2011a).

MiR-33 is an evolutionarily conserved miRNA that is located within intron 16 of the gene encoding sterol-regulatory element-binding protein-2 (SREBP-2) and is co-transcribed with its host gene (Rayner et al, 2010). Moreover, miR-33 targets the sterol transporters ABCG1 and ABCA1 (ATP-binding cassette transporters) and decreases the efflux of cholesterol to high-density lipoproteins (HDL) which are involved in the removal of excess cholesterol from the body (Horie et al, 2010; Rayner et al, 2010). Silencing of miR-33 levels *in vivo* increased circulating HDL levels and promoted reverse cholesterol transport (RCT) suggesting a new therapy strategy to treat atherosclerosis and other metabolic diseases (Rayner et al, 2011a, b). In a recent report, Cirera-Salinas et al (2012) found that miR-33 is involved in cell proliferation and cell cycle progression suggesting an essential role for miR-33 in regulating hepatocyte proliferation during human liver regeneration.

» …miR-33 regulates RCT through modulation of HDL metabolism (via ABCA1) and bile metabolism (via ABCB11 and ATP8B1).«

**Figure 1:**
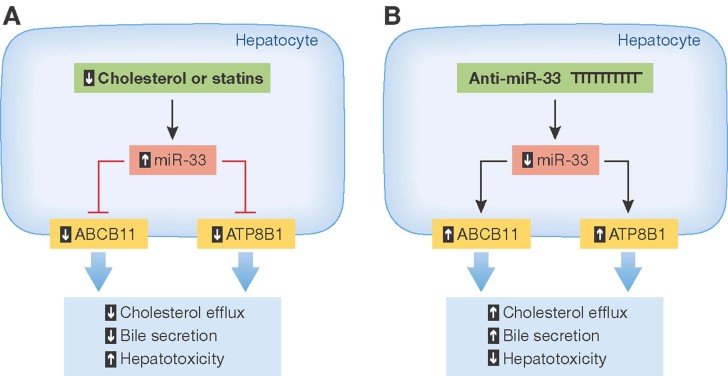
Silencing of miR-33 rescues the statin- and diet-induced liver damage Intracellular low cholesterol levels or statin treatment induce the expression of miR-33 and reduce the expression of the sterol transporters ABCB11 and ATP8B1 leading to a decrease in cholesterol efflux and bile secretion. This results in enhanced hepatotoxicity.Silencing miR-33 using LNA oligonucleotides reduces hepatic miR-33 levels resulting in elevated expression of the target genes ABCB11 and ATP8B1. This leads to an increase in cholesterol efflux and bile flow and prevents hepatotoxicity.

In this issue of *EMBO Molecular Medicine*, Allen et al (2012) report a role for miR-33 during the modulation of hepatic bile metabolism by reducing the expression of specific sterol transporters in the canalicular membrane of hepatocytes. The authors show that two of these transporters, ATP-binding cassette, sub-family B member 11 (ABCB11) and ATPase, aminophospholipid transporter, class I, type 8B, member 1 (ATP8B1) are direct targets of miR-33. To investigate the role of miR-33 *in vivo*, chow-fed mice were injected with saline, scrambled and anti-miR-33 locked nucleic acid (LNA) oligonucleotides. The volume of bile recovered from gallbladders was twofold increased in anti-miR-33 injected animals compared to controls after 1 week. Further, the expression of hepatic *Abcb11* and *Atp8b1* were significantly increased in mice injected with anti-miR-33 oligonucleotides. In contrast, overexpression of miR-33 using an miR-33-encoding adenovirus resulted in a decrease of ABCB11 and ATP8B1 expression in mouse and human hepatocytes. In general, a lithogenic diet (21% fat, 1.25% cholesterol and 0.5% cholate) in mice is linked to disrupted bile homeostasis and the development of cholestasis after few weeks (Khanuja et al, 1995). Allen et al found a significant reduction in hepatic miR-33 expression in mice fed a lithogenic diet. Under diet-induced cholestasis condition, mice showed increased miR-33 expression in the livers. Further, the volume of bile was 45% smaller than control mice indicating hepatic bile retention. The analysis of the liver content resulted in a significant increase in hepatic bile acids, total cholesterol and esterified cholesterol in mice overexpressing miR-33, whereas the bile content revealed a significant decrease in bile acids and a slight increase in cholesterol. The amount of triglycerides was reduced in the livers of mice overexpressing miR-33. The authors speculate that the decrease in liver triglycerides is due to reduced hepatic expression of *Fasn* in miR-33 overexpressing mice. However, the impact of miR-33 on lipid metabolism *in vivo* will require further investigations. Of note, the expression of *Abcb11* and *Atp8b1* was significantly decreased in mice receiving miR-33 vectors. In line, mice chow-fed showed also a reduced expression of both transporters *Abcb11* and *Atp8b1* indicating that miR-33 can also regulate basal expression levels of these transporters *in vivo* under normal diet circumstances. Interestingly, the sterol transporters *Abcg5* and *Abcg8* were similarly significantly decreased in adenoviral-mediated overexpressing miR-33 mice fed the lithogenic diet. Because the human and murine ATP-binding cassette sub-family G member 5 (ABCG5) and ATP-binding cassette sub-family G member 8 (ABCG8) genes are not direct targets of miR-33 and no repression was observed after overexpressing miR-33 in mouse primary hepatocytes and human hepatocytes, the authors argue that this could be the result of off-target effects using the adenoviral approach. Indeed, mice deficient in ABCB11 showed reduced hepatic levels of ABCG5 and ABCG8 fed a lithogenic diet (Wang et al, 2003). However, the underlying mechanism involved in this reduced expression of *Abcg5/8* needs to be elucidated. The authors also investigated the effect of miR-33 on RCT, which mediates the mobilization of excess cholesterol from cells back to the liver for excretion to the bile and finally the faeces (Wang & Rader, 2007). Consistently, ABCA1 has been shown to be involved in RCT (Wang & Rader, 2007). Furthermore, systemic silencing of miR-33 in mice resulted in an enhanced RCT to the plasma, liver and faeces due to increased ABCA1-dependent cholesterol efflux (Rayner et al, 2011b). Using radiolabelled cholesterol the authors investigated the physiological impact of miR-33 on the mobilization of extrahepatic cholesterol into the bile. Systemic silencing of miR-33 led to an increased amount of labelled cholesterol in plasma and increased labelled sterols in the bile. Hence, miR-33 regulates RCT through modulation of HDL metabolism (via ABCA1) and bile metabolism (via ABCB11 and ATP8B1) (Fig. 1).

» …promising new approach to treat cholestatic syndromes due to statin- or diet-induced liver damage and other metabolic disorders by manipulating hepatic miR-33«

The finding that administration of simvastatin and atorvastatin induced miR-33 expression and decreased mRNA levels of miR-33 targets in hepatocytes is an important issue for current and future clinical applications. Accordingly, the effect of statins and lithogenic diet on mice was examined showing that simvastatin increased miR-33 levels in a dose-dependent way in chow-fed mice. Notably, a dose-dependent lethality effect of statins was observed in mice after switching to the lithogenic diet. Further, this statin-induced hepatotoxicity led to a decrease in cholesterol and reduced *Abcb11* and *Atp8b1* mRNAs levels of 40%. Systemic silencing of miR-33 using LNA oligonucleotides rescued the statin- and diet-induced phenotype. Treatment with anti-miR-33 oligonucleotides resulted in reduced miR-33 expression of 40% and further led to a significant increased expression of *Atp8b1* but not *Abcb11*. The authors argued that the expression of *Abcb11* is already at the maximal expression level in these livers because of the diet-induced activation of FXR. However, the differences in the expression of these genes make it difficult to interpret. The findings of Allen et al (2012) demonstrate that manipulation of miR-33 might be a new therapeutic approach for the treatment of cholestatic syndromes. Indeed, miRNA based therapeutic approaches have been convincingly shown to be successful in many vascular and cardiovascular diseases (Thum, 2012). Administration of statins to lower cholesterol levels result in several side effects of hepatotoxicity such as cholestasis. So far, no drug-mediated approach is available to treat statin-induced liver damage. A combined therapy of statins and anti-miR-33 oligonucleotides may probably prevent statin-induced hepatotoxicity. However, more studies about this novel therapeutic approach are needed including studies in large animals such as primates to bring this approach to the clinic.

Nevertheless, some issues still remain. The study does not consider that the human genome encodes for two miR-33 isoforms in the Srebp genes. Mice do not encode for miR-33b from an intron of SREBP-1 (Moore et al, 2011; Rayner et al, 2011a). Therefore, more studies would be helpful to investigate the importance of SREBP-1-derived miR-33b on bile metabolism. Furthermore, the question which specific transporter(s) contributes to the effect of anti-miR-33 treatment on RCT or to statin-induced miR-33-dependent hepatotoxicity has not been taken into account and remains to be determined. Additional studies using mice deficient for each of these transporters might provide more mechanistic insights. Despite these issues, the study opens up a promising new approach to treat cholestatic syndromes due to statin- or diet-induced liver damage and other metabolic disorders by manipulating hepatic miR-33 expression.
